# ‘Help for Hay Fever’, a goal-focused intervention for people with intermittent allergic rhinitis, delivered in Scottish community pharmacies: study protocol for a pilot cluster randomized controlled trial

**DOI:** 10.1186/1745-6215-14-217

**Published:** 2013-07-15

**Authors:** Terry Porteous, Sally Wyke, Sarah Smith, Christine Bond, Jill Francis, Amanda J Lee, Richard Lowrie, Graham Scotland, Aziz Sheikh, Mike Thomas, Lorraine Smith

**Affiliations:** 1 Division of Applied Health Sciences, University of Aberdeen, Polwarth Building, Foresterhill, Aberdeen AB25 2ZD, UK; 2 Institute of Health and Wellbeing, College of Social Sciences, Room 227, University of Glasgow, 27 Bute Gardens, G12 8RS Glasgow, UK; 3 School of Health Sciences, City University London, Northampton Square, EC1V 0HB London, UK; 4 Long Term Conditions and Research Team, Pharmacy and Prescribing Support Unit, Queens Park House, Victoria Infirmary, Glasgow G42 9TY, UK; 5 Allergy and Respiratory Research Group, Centre for Population Health Sciences, Medical School, The University of Edinburgh, Doorway 3, Teviot Place, Edinburgh EH8 9AG, UK; 6 Primary Care Research, Aldermoor Health Centre, University of Southampton, Aldermoor Close, Southampton SO16 5ST, UK; 7 Faculty of Pharmacy, The University of Sydney, Building A15, New South Wales 2006, Australia

**Keywords:** Respiratory, Allergy, Community pharmacy, Self-care

## Abstract

**Background:**

Despite the availability of evidence-based guidelines for managing allergic rhinitis in primary care, management of the condition in the United Kingdom (UK) remains sub-optimal. Its high prevalence and negative effects on quality of life, school performance, productivity and co-morbid respiratory conditions (in particular, asthma), and high health and societal costs, make this a priority for developing novel models of care. Recent Australian research demonstrated the potential of a community pharmacy-based ‘goal-focused’ intervention to help people with intermittent allergic rhinitis to self-manage their condition better, reduce symptom severity and improve quality of life. In this pilot study we will assess the transferability of the goal-focused intervention to a UK context, the suitability of the intervention materials, procedures and outcome measures and collect data to inform a future definitive UK randomized controlled trial (RCT).

**Methods/Design:**

A pilot cluster RCT with associated preliminary economic analysis and embedded qualitative evaluation. The pilot trial will take place in two Scottish Health Board areas: Grampian and Greater Glasgow & Clyde. Twelve community pharmacies will be randomly assigned to intervention or usual care group. Each will recruit 12 customers seeking advice or treatment for intermittent allergic rhinitis. Pharmacy staff in intervention pharmacies will support recruited customers in developing strategies for setting and achieving goals that aim to avoid/minimize triggers for, and eliminate/minimize symptoms of allergic rhinitis. Customers recruited in non-intervention pharmacies will receive usual care. The co-primary outcome measures, selected to inform a sample size calculation for a future RCT, are: community pharmacy and customer recruitment and completion rates; and effect size of change in the validated mini-Rhinoconjunctivitis Quality of Life Questionnaire between baseline, one-week and six-weeks post-intervention. Secondary outcome measures relate to changes in symptom severity, productivity, medication adherence and self-efficacy. Quantitative data about accrual, retention and economic measures, and qualitative data about participants’ experiences during the trial will be collected to inform the future RCT.

**Discussion:**

This work will lay the foundations for a definitive RCT of a community pharmacy-based ‘goal-focused’ self-management intervention for people with intermittent allergic rhinitis. Results of the pilot trial are expected to be available in April 2013.

**Trial registration:**

Current Controlled Trials ISRCTN43606442

## Background

Allergic rhinitis is a long-term condition with a high prevalence (estimated at 26% in the UK [1]) that has increased substantially in recent years [2]. Allergic rhinitis has a significant negative impact on quality of life (QOL) [3], productivity [4], school performance [5] and health care costs [6,7]. Co-morbidity of allergic rhinitis with asthma is common and is associated with poor asthma outcomes [8-10]. Allergic rhinitis is a common presenting condition in community-based health care, with community pharmacies often representing the first point-of-contact with health services [11].

Evidence-based guidelines for medical practitioners and pharmacists have been introduced [12-14]. Two RCTs of general practitioner (GP) guidelines for allergic rhinitis have demonstrated improvements in patients’ allergic rhinitis-related outcomes when guidelines are implemented, either with [15] or without [16] specific training for primary care staff. Nevertheless, primary care management of allergic rhinitis is still sub-optimal in the UK [11,17,18] suggesting that significant avoidable morbidity still exists. Care of long-term conditions is increasingly shifting to the community, opening up new opportunities for pharmacists to effect sustainable changes in care delivery. Community pharmacists are well placed to recognize and recommend treatment for allergic rhinitis, particularly for intermittent (seasonal) and mild cases [13], however, our searches of the literature failed to identify any pharmacy-based interventions for allergic rhinitis in the UK.

Two previous Australian studies suggested that a community pharmacy-delivered goal-focused approach has the potential to improve outcomes for patients with allergic rhinitis [19-21]. Interpretation of the Australian research was complicated by the lack of true comparison populations, but despite this, the findings suggest that the intervention could potentially improve allergic rhinitis management. We propose undertaking a definitive RCT in the UK, but prior to this, in accordance with Medical Research Council guidance on the development of complex interventions [22], the intervention must be piloted to test transferability of the methods and measures used in Australia, and derive estimates of effect sizes, participation rates and completion rates that would be needed to fully evaluate a similar intervention in the UK. Additionally, our pilot trial will address limitations of the Australian research [19-21] including lack of a true comparison and collection of data for a comprehensive economic evaluation.

## Study design

The ‘Help for Hay Fever’ study is a pilot cluster RCT with associated preliminary economic analysis and an embedded qualitative study involving semi-structured follow-up interviews with a sub-sample of participants (community pharmacy staff and customers). Trained staff (pharmacists and/or pharmacy assistants) will recruit eligible customers with intermittent allergic rhinitis; the intervention will be delivered immediately after recruitment. Patient-reported outcomes will be measured at baseline and again at one week and six weeks after recruitment using a validated, disease-specific instrument [23]. The trial will compare outcomes in people who receive the intervention (intervention group) with those receiving usual care. In addition, we will collect quantitative data about accrual, retention and economic measures, and qualitative data about participants’ experiences during the trial, to inform a future full scale trial. Study materials for this pilot trial will be based on those used in the Australian studies [19-21].

### Ethical considerations

This pilot trial will be conducted in accordance with NHS research governance requirements and Good Clinical Practice Guidelines [24]. All necessary research ethics and NHS Research and Development approvals have been obtained from the North of Scotland Research Ethics Service and from NHS Research Scotland Permissions Coordinating Center respectively, prior to starting recruitment.

### Aims and research questions

The aims are to adapt and pilot a community pharmacy-delivered, goal-focused intervention for the self-management of intermittent allergic rhinitis in Scotland, based on previous research in Australia, and to collect data to inform a future definitive Scottish RCT.

**Figure 1:**
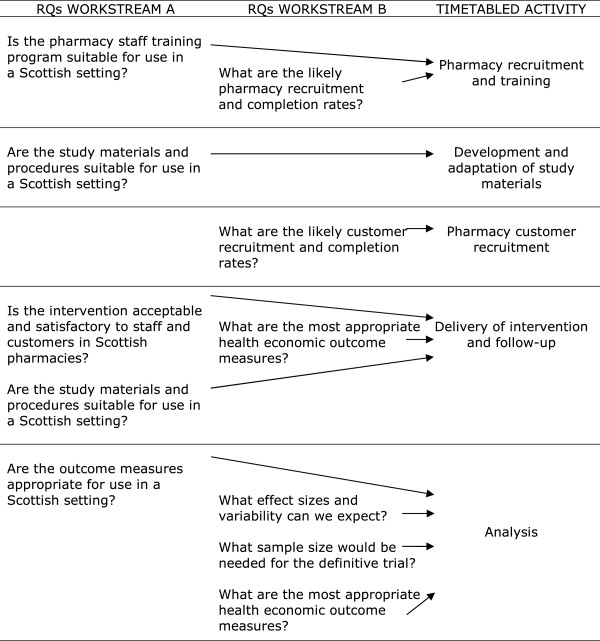
Relationships between the timetabled activities and the research questions (RQ).

The research questions below will be addressed by two parallel work streams (A and B) which will run simultaneously. The relationships between the timetabled activities and the research questions are illustrated in Figure 1.

A. Questions about transferability of methods and measures used previously in the Australian studies:

RQ:A1. Is the pharmacy staff training program suitable for use in a Scottish setting?

RQ:A2. Is the allergic rhinitis intervention acceptable and satisfactory to pharmacy staff delivering and customers receiving the service in Scottish pharmacies?

RQ:A3. Are the study materials and procedures suitable for use in a Scottish setting?

RQ:A4. Are the outcome measures appropriate for use in a Scottish setting?

B. Questions to inform a future definitive evaluation by RCT:

RQ:B1. What are the likely pharmacy recruitment rates and completion rates?

RQ:B2. What are the likely customer recruitment rates and completion rates?

RQ:B3. What effect sizes and variability can we expect?

RQ:B4. What sample size would be needed for the definitive trial?

RQ:B5. What are the most appropriate health economic outcome measures?

### Participants and setting

#### Eligibility of community pharmacies entering the trial

All community pharmacies in NHS Grampian and NHS Greater Glasgow & Clyde will be eligible to participate. The only condition of inclusion will be that pharmacy staff (at least one pharmacist and one pharmacy assistant) are available to attend training if randomized to the intervention group.

#### Eligibility of pharmacy customers entering the trial

Customers presenting with active symptoms of intermittent allergic rhinitis at participating community pharmacies will be eligible to take part. Each community pharmacy will recruit 12 participants from customers attending the pharmacy, aged ≥ 18 years and able to communicate in English. Customers eligible for inclusion will be those with active symptoms of allergic rhinitis, that is, respiratory/ocular symptoms consistent with intermittent allergic rhinitis as defined by ARIA (Allergic Rhinitis and its Impact on Asthma) guidelines [12]. They will have had two or more relevant symptoms for at least an hour on fewer than four days per week, or for fewer than four consecutive weeks. Those symptoms will include: runny or blocked nose (both sides); itching nose and/or throat; itching, sore and/or red eyes; and sudden bouts of sneezing. Exclusion criteria are: pregnancy, terminal illness, and current or recent involvement (within the past two years) in an allergic rhinitis study.

### Sampling and recruitment

**Figure 2:**
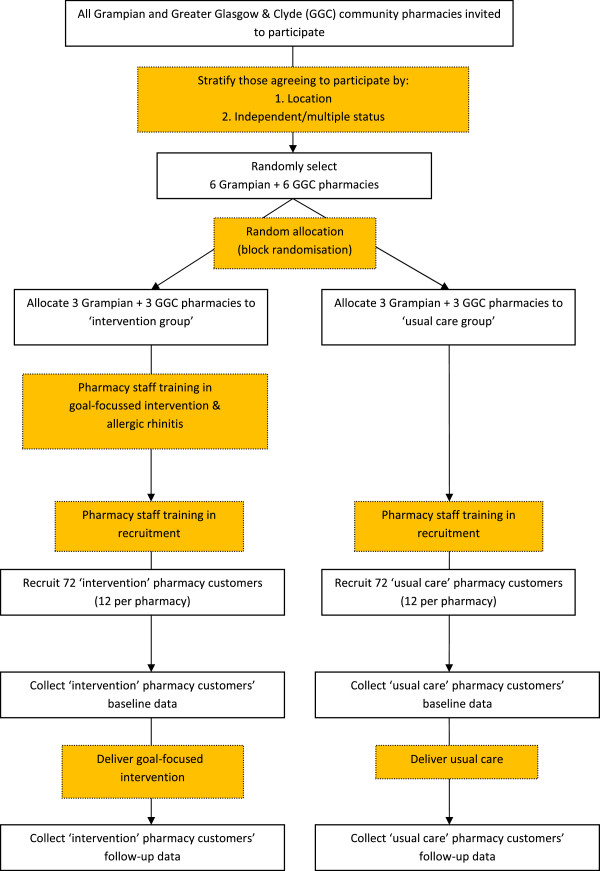
Flowchart of recruitment.

Figure 2 shows a flowchart of the recruitment process.

#### Community pharmacies

An Email advertising the study, followed by a mailed invitation to participate, will be sent to all community pharmacies in the Grampian and Greater Glasgow & Clyde Health Board areas (n = 130 and 314 respectively). A stratified random sample of participants will be selected from a list of all eligible community pharmacies responding positively. Participating community pharmacies will receive an honorarium of £10/ patient recruited at the end of the recruitment period.

#### Pharmacy customers

Potential participants will be identified when they ask for advice about symptoms, or request a named treatment, or present a prescription for treatment of intermittent allergic rhinitis. Eligible customers will be provided with a Participant Information Sheet explaining what the study involves, advised that they may withdraw at any time without prejudice to the quality of care they will receive in the community pharmacy, and given the opportunity to ask questions about the study. Those agreeing to participate will be asked to give written consent. To enhance follow-up rates, as recommended in a recent Cochrane review [25], participants will receive a gift voucher to the value of £10 as a thank you for taking part.

### Randomization

The unit of randomization will be the community pharmacy. Using block randomization with a block size of six, an independent statistician from the Medical Statistics Team, Division of Applied Health Sciences, University of Aberdeen, will select a random sample of 12 community pharmacies, stratified by location (Grampian or Greater Glasgow & Clyde) and by pharmacy status (independent/small multiple/national multiple) from those responding positively to the mailed invitation. Stratification by these variables aims to account for any potential variation resulting from urban/rural characteristics, pharmacy staff and typical customers of included community pharmacies. Six pharmacies will be allocated to each of the intervention and usual care groups.

### Intervention

#### Intervention group

With the aim of enhancing replicability of the intervention for a future RCT, we have applied a reliable and valid taxonomy of behavior change techniques (BCTs) from recent UK-based research [26,27] to produce a clearer specification of its components. Two authors (JF and LS) independently specified each element of the intervention and defined them using this taxonomy. The BCTs identified by each author were compared and any discrepancies discussed until consensus was reached. The core BCTs identified in the intervention are captured by four of the taxonomy’s 16 clusters: *Goals and planning* (specific BCTs: goal-setting (outcome); goal-setting (behavior); problem solving; action planning), *Natural consequences* (specific BCT: information about health consequences), *Regulation* (specific BCT: pharmacological support), and *Feedback and Monitoring* (specific BCTs: self-monitoring of behavior; self-monitoring of outcome(s) of behavior). Additional BCTs that may or may not be used, depending on the personalized elements of the intervention, are part of the clusters of: *Regulation* (BCT: social support), *Antecedents* (BCTs: restructuring the physical environment; restructuring the social environment) and *Associations* (BCT: prompts/cues). Table 1 shows the output from this exercise and how the BCTs are operationalized in the ‘Help for Hay Fever’ intervention.

**Table 1:** **Specifying the intervention using a taxonomy of behavior change techniques (BCTs)**[22,23]

**Core elements of the intervention**		
**Label**	**Definition**	**How is it operationalized in the ‘Help for Hay Fever’ study**?
*Goals and planning*		
Goal-setting outcome	Set or agree a goal defined in terms of a positive outcome of wanted behavior.	Set a pre-determined outcome goal: ‘*eliminate*/*minimize symptoms*.’
Goal-setting behavior	Set or agree a goal defined in terms of the behavior to be achieved.	Set a pre-determined behavior goal: ‘*avoid*/*minimize triggers*’.
Problem solving	Analyze factors influencing the behavior and generate or select strategies that include overcoming barriers and/or increasing facilitators.	Identify specific barriers to achieving optimum management of hay fever, for example, not taking medication regularly, and suggest personalized strategies to overcome this, for example, provide prompts/cues for medication adherence.
Action planning	Prompt detailed planning of performance of the behavior.	Plan personalized strategies to achieve the two specified goals (avoid/minimize triggers and eliminate/minimize symptoms) and complete a personalized goals card:
		a) Focus on personal symptoms and symptom triggers and help the person to develop specific behavioral strategies to achieve the goals.
		b) Negotiate the strategies the person would like to use to control the symptoms/triggers and ask them to write these on the goals card.
*Natural consequences*		
Information about health consequences	Provide information about health consequences of performing the behavior.	Present the likelihood of an improved quality of life, reduced symptom severity, better productivity.
*Regulation*		
Pharmacological support	Provide, or encourage the use of or adherence to, drugs to facilitate behavior change.	Advise on hay fever medications and encourage medication adherence.
*Feedback and monitoring*		
Self-monitoring of behavior	Establish a method for the person to monitor and record their behavior(s) as part of a behavior change strategy.	Ask the person to record daily whether or not they have taken their hay fever medication, using a diary booklet provided.
Self-monitoring of outcome(s) of behavior	Establish a method for the person to monitor and record the outcome(s) of their behavior as part of a behavior change strategy.	Ask the person to record daily their symptom severity score using a diary booklet provided.
**Personalized elements of the intervention**		
*Regulation*		
Social support (practical)	Advise on, arrange, or provide practical help (for example, from friends, relatives, colleagues, ‘buddies’ or staff) for performance of the behavior.	Provide if appropriate, based on discussion with customer about how social contacts might facilitate trigger avoidance for example, ask a friend, relative or neighbor to cut the grass for them.
*Antecedents*		
Restructuring the physical environment	Change, or advise to change the environment in order to facilitate performance of the wanted behavior or create barriers to the unwanted behavior (other than prompts/cues, rewards and punishments).	Provide if appropriate, based on discussion with customer about any specific hay fever triggers.
		a) Changes to physical environment might include moving indoor pot plants; removing visible mould with bleach/ anti-mould cleaner; closing windows.
Restructuring the social environmen**t**	Change, or advise to change the social environment in order to facilitate performance of the wanted behavior or create barriers to the unwanted behavior (other than prompts/cues, rewards and punishments).	b) Changes to social environment might include avoiding social occasions that trigger symptoms, for example, outdoor picnics.
*Associations*		
Prompts/cues	Introduce or define environmental or social stimulus with the purpose of prompting or cueing the behavior.	Provide if appropriate, based on discussion with customer about any barriers to the target behavior, for example, leaving hay fever medication in an obvious place to remind people to take it in the morning (beside car keys, next to toothbrush, and so on).

#### Community pharmacy staff

Recruitment and training of pharmacy staff will take place over a period of three months. One pharmacist and at least one pharmacy assistant from each of the six intervention pharmacies will attend a three hour training workshop; one workshop will be held in each of the Grampian and Greater Glasgow & Clyde Health Board areas. The training program will be a ‘refresher’ about current best practice in allergic rhinitis and the ARIA guidelines for pharmacy management of allergic rhinitis [13]. The workshop will include training in self-management theory, the use of goal-setting as a behavior change technique and participant recruitment (including taking consent). At the end of the workshop, participants will take part in a role play scenario, allowing them the opportunity to experience the process of delivering the intervention. The training aims to motivate pharmacy staff, for example, by giving them the confidence to deliver the intervention and emphasizing how the intervention could improve patients’ allergic rhinitis-related QOL. As soon as possible after training, a researcher will visit each intervention community pharmacy to deliver the necessary study materials. During this visit, the researcher will go through the training materials once more with at least one staff member who had attended the training evening. Pharmacy staff will then be given the opportunity to ask any remaining questions about the study before commencing recruitment.

#### Community pharmacy customers

Recruitment of pharmacy customers will take place over a period of four months. Pharmacy customers who are eligible and willing will receive the one-off intervention that was developed in Australia. In addition to advising customers about treatment and management of allergic rhinitis, pharmacy staff will provide them with a card containing two pre-determined goals (‘*Avoid*/*minimize hay fever triggers*’ and ‘*Eliminate*/*minimize hay fever symptoms*’). This card provides space for participants to record (in writing) their personal allergic rhinitis triggers and symptoms; pharmacy staff will support them in formulating action plans for achieving these two goals. Participants will also receive printed information to take home about allergic rhinitis symptoms, triggers and general information.

#### Usual care group

Pharmacy staff randomized to the usual care arm will receive no training in allergic rhinitis. A researcher will visit each of the six usual care community pharmacies to deliver the study materials, and to train staff in recruitment, consent taking and use of the data collection materials. Customer recruitment will begin immediately after this visit. Recruitment of pharmacy staff and customers in the usual care group will take place contemporaneously with the intervention group.

### Outcome measures and instruments

#### Quantitative outcomes

The co-primary outcomes are:

• community pharmacy and pharmacy customer recruitment and completion rates;

• effect size and variability of change in allergic rhinitis-related QOL.

The decision about whether or not to undertake a RCT will be informed by pharmacy and customer recruitment and completion rates, and the sample size calculation informed by the estimated effect size of the mini-Rhinoconjunctivitis Quality of Life Questionnaire (mini-RQLQ). This is a validated and widely used disease-specific health status instrument, the psychometric properties and ‘minimally clinically important differences’ of which are well described [23]. The domains covered by the mini-RQLQ are: activities; practical problems; nose symptoms; eye symptoms; and other symptoms. Although this pilot study is not powered to detect any statistically significant between-group changes in mini-RQLQ scores, the decision to proceed to full trial will be informed by any trend in customer benefit of the intervention; it is unlikely that a RCT will be undertaken if no improvement or a deterioration in mini-RQLQ score between groups is observed.

Our selected secondary outcome measures represent a range of clinical indicators, social functioning and economic outcomes that are relevant to allergic rhinitis [23,28]:

• symptom severity using a 12-item scale from Spector 2003 [29] adapted for use in the Australian studies.

• productivity using the six-item Work Productivity and Activity Impairment Questionnaire: Allergy Specific [30,31] .

• medication adherence using the five-item Medication Adherence Reporting Scale [32].

• self-efficacy using the Lorig six-item generic instrument [33] adapted for use in the Australian studies.

• health state utilities and quality adjusted life years based on responses to the EQ-5D [34].

• pharmacy and health service costs.

• costs to patients and production effects.

A costing exercise will be undertaken to estimate the direct costs of the intervention to pharmacy staff and customers, including resources used in training, as well as indirect production effects. Data will also be collected about customers’ allergic rhinitis-related contacts with health services and medication use during the six-week follow-up period, both measured by patient self-report.

Data will be collected from both intervention and usual care customers as described above. We recognize that, as is the case in any trial collecting self-report data, the action of completing the data collection instruments may in itself contribute to changes in allergic rhinitis management. However, we anticipate that any such effect is likely to be similar in both intervention and usual care groups. Any between group differences are therefore unlikely to be attributable to any ‘Hawthorne effect’.

In addition to the above, data concerning the training workshops will be collected from participating community pharmacy staff in the intervention group. This data will inform the training for a future RCT:

• training workshops will be evaluated using a questionnaire based on the one used previously in Australia [35]. It will cover satisfaction with and acceptability of the training content, and the way in which it is delivered.

• a multiple choice questionnaire, developed for this study, will measure knowledge of allergic rhinitis management and treatment, before and after the training workshop.

#### Embedded qualitative study

Six weeks after recruitment, semi-structured telephone exit interviews will be undertaken with a purposively selected sub-sample of intervention customers (three from each pharmacy, n = 18) representing a range of age, gender and self-reported symptom severity scores. The interview will elicit views on: acceptability, experiences of, and satisfaction with the intervention; what worked best for them; and acceptability of the measures used. A quantitative question will be also be included to determine the maximum amount of money that clients in the intervention group would have been willing to pay for the service they received. The interview schedule will be informed by the findings of the previous Australian research.

Following completion of customer recruitment in community pharmacies, semi-structured interviews with at least one participating member of staff from each intervention pharmacy will collect qualitative data about: their experiences of implementing the intervention; perceived benefits of and barriers to introducing it into usual practice; and general issues around acceptability of, and satisfaction with, the intervention. For the economic analysis, we will assess the potential willingness of intervention pharmacy staff to provide the intervention under existing contract arrangements or through separate funding streams. A question will be also be included to determine any additional fees that pharmacies would demand in order to provide the service to the NHS or private paying clients. The interview schedule will be informed by the findings of the previous Australian research.

### Data collection

Copies of our data collection instruments can be obtained on application to the authors.

Using a ‘recruitment log’, staff in participating pharmacies who are responsible for recruitment will keep a tally of the number of customers approached and record the number accepting, the number declining, and the number excluded because they did not meet the inclusion criteria. Data from the recruitment log will provide denominators for calculating recruitment and completion rates at the end of the study period, and allow realistic estimation of the period required for recruitment.

All eligible customers in both intervention and usual care groups who agree to participate will complete a baseline questionnaire in the pharmacy, collecting data about demographic characteristics and allergic rhinitis-related outcome data including: QOL, symptom severity, productivity, medication adherence and self-efficacy. Additional data about participants’ allergic rhinitis history and their contact details will be collected by pharmacy staff using forms provided for that purpose. To inform the economic analysis, the staff member responsible for recruiting individual customers will record the time taken per customer, to advise about allergic rhinitis management and (where appropriate) deliver the intervention. All data collected in the community pharmacy will be returned to the research team on the day of recruitment to allow researchers to follow up recruited customers in a timely fashion.

Each day in the week following recruitment, using a diary booklet provided for the purpose, participants will record an allergic rhinitis severity score (‘On a scale of 1 to 10, I think my symptoms have been…’) and their adherence to allergic rhinitis medication (‘I have taken my hay fever medication’; YES/NO). During that first week, a copy of the same questionnaire used at baseline will be mailed to all participants. This will be completed one week after recruitment and returned, together with their completed diary booklet (symptom severity and medication adherence) in a reply-paid envelope provided. Six weeks after recruitment, all participants will receive another postal questionnaire to collect a sub-set of the outcome data (QOL and symptom severity), and additional data to inform a future economic evaluation of the intervention (allergic rhinitis-related contacts with health services and quantities of allergic rhinitis medication used over the six week period). Non-responders will receive one telephone reminder and the opportunity to complete the questionnaire by telephone if they prefer this to returning the postal questionnaire.

As described above, semi-structured interviews will be used to collect data about participants’ experiences of the intervention. These will be conducted by telephone for pharmacy customers and either by telephone or face-to-face for pharmacy staff, as preferred by the interviewee. Data will be recorded manually by the researcher using written field notes, recorded on a data collection form developed for this purpose.

### Post-recruitment retention strategies

All participating community pharmacies will be contacted by telephone within four weeks of commencing customer recruitment to ensure that they are experiencing no problems with the study procedures. Support from the study researcher will be offered to pharmacy staff to resolve any such problems. A newsletter will be sent to all pharmacies during the intervention period reminding staff of the study procedures, and giving feedback about customer recruitment to date and tips on how to enhance recruitment. These tips will be a reminder of the strategies suggested during training, but will also be informed by feedback from the routine telephone contacts made with pharmacy staff in the four weeks after customer recruitment commences. Recruitment of customers will be monitored for each community pharmacy throughout the four-month recruitment period. Pharmacies that are not reaching target recruitment will be offered one-to-one support by the study researcher. If the problem continues into the second month of the recruitment period, two solutions will be considered: pharmacies that have successfully reached their recruitment target will be asked to continue recruiting more customers, and/or additional community pharmacies will be recruited and trained from the list of those expressing an interest to the original mailed invitation to participate.

Recruited pharmacy customers whose completed one-week questionnaire has not been received by the research team three weeks post-recruitment, will receive a reminder from the study researcher. Reminders will be issued by telephone or email as indicated in the customers’ contact details provided at recruitment. Attempts to contact non-responders will continue until contact is made or for a maximum of one week. A similar process will be followed for the six-week questionnaire.

### Data management and analysis

#### Quantitative analysis

Data will be entered into Statistical Package for the Social Sciences (Version 20.0, IBM, UK Head Office, IBM United Kingdom Limited, PO Box 41, North Harbour, Portsmouth, Hampshire, PO6 3AU.) for analysis including: patient level descriptive statistics; inter-group comparisons of intervention and usual care groups (for example, cluster adjusted chi-squared test); and intra-group comparisons (for example, cluster adjusted McNemars test) based on the generalized estimating equations (GEE) approach. Effect size estimates and the intra-cluster correlation coefficient (ICC) will be calculated to inform the sample size calculation for a definitive RCT.

#### Economic analysis

Pharmacy staff’s time inputs will be valued using standard unit costs incorporating salaries, oncosts, capital and overheads. Out-of-pocket patient costs (for example, medications) and productivity will be estimated using questions from the baseline and follow-up questionnaires; medications will be costed using market prices and productivity will be valued using age/sex specific average gross wage rates. The feasibility of using the EQ-5D to track changes in health related QOL - to enable the estimation of quality adjusted life years (QALYs) - will also be assessed using data from this instrument in the patient questionnaires. A cost-utility analysis will be undertaken, adopting both a health service and societal perspective.

#### Qualitative analysis

Qualitative data will be stored and organized using Microsoft Excel (Microsoft Limited, Microsoft Campus, Thames Valley Park, Reading, Berkshire, RG6 1WG, United Kingdom.), and analyzed using thematic descriptive analysis based on the research questions (RQs A1-A4), but alert to any unexpected experiences and views. The field notes collected by the researcher during post-intervention interviews will be transcribed into Excel. These data will be grouped together by themes emerging and reported descriptively.

### Sample size considerations

Since this is a pilot RCT, no formal sample size calculation is necessary [36] although sufficient numbers are required in each pharmacy to estimate effect sizes and give a reliable ICC that will inform a future full evaluation. Hence, we aim to retain at least 10 pharmacies (five per arm) with 10 patients in each pharmacy. To allow for attrition, we initially aim to recruit 12 pharmacies (six per arm) each of which will be asked to recruit 12 patients (that is, a total sample size of 144 patients). In the event of insufficient recruitment of pharmacy customers, or if any pharmacies drop out following recruitment, additional pharmacies from the list of those expressing an interest in participating will be selected to boost recruitment numbers.

The sample sizes for the two qualitative components (sub-sample of intervention customers (n = 18) and intervention pharmacy staff (n = 6)) are pragmatic and are considered sufficient to provide some feedback on the delivery and acceptability of the intervention.

## Trial status

The study began in April 2012. At the time of submission, 12 community pharmacies have been recruited. Pharmacy staff from the six intervention pharmacies (three in each of Grampian and Greater Glasgow & Clyde Health Board areas) have been trained and recruitment of community pharmacy customers commenced at the end of June 2012. Pharmacy customer recruitment is ongoing and monthly targets are being met.

## Abbreviations

ARIA: Allergic Rhinitis and its Impact on Asthma; BCT: Behavior change technique; BCTs: Behavior change techniques; GEE: Generalized estimating equation; GP: General Practitioner; ICC: Intra-cluster correlation coefficient; mini-RQLQ: mini-Rhinoconjunctivitis Quality of Life Questionnaire; QALYs: Quality adjusted life years; QOL: Quality of life; NHS: National Health Service; RCT: Randomized controlled trial; RQ: Research questions; UK: United Kingdom.

## Competing interests

The authors declare that they have no competing interests.

## Authors’ contributions

All authors participated in the design of the trial, are members of the project steering group, and contributed to, read and approved the final manuscript.
